# Sequential inference as a mode of cognition and its correlates in fronto-parietal and hippocampal brain regions

**DOI:** 10.1371/journal.pcbi.1005418

**Published:** 2017-05-09

**Authors:** Thomas H. B. FitzGerald, Dorothea Hämmerer, Karl J. Friston, Shu-Chen Li, Raymond J. Dolan

**Affiliations:** 1 The Wellcome Trust Centre for Neuroimaging, University College London, London, United Kingdom; 2 Max Planck – UCL Centre for Computational Psychiatry and Ageing Research, London, United Kingdom; 3 Department of Psychology, University of East Anglia, Norwich Research Park, Norwich, Norfolk, United Kingdom; 4 Chair of Lifespan Developmental Neuroscience, Department of Psychology, TU Dresden, Dresden, Germany; 5 Institute of Cognitive Neuroscience, University College London, London, United Kingdom; 6 Institute of Cognitive Neurology and Dementia Research, Otto-von-Guericke University Magdeburg, Magdeburg, Germany; 7 Center for Lifespan Psychology, Max Planck Institute for Human Development, Berlin, Germany; Brain and Spine Institute (ICM), FRANCE

## Abstract

Normative models of human cognition often appeal to Bayesian filtering, which provides optimal online estimates of unknown or hidden states of the world, based on previous observations. However, in many cases it is necessary to optimise beliefs about sequences of states rather than just the current state. Importantly, Bayesian filtering and sequential inference strategies make different predictions about beliefs and subsequent choices, rendering them behaviourally dissociable. Taking data from a probabilistic reversal task we show that subjects’ choices provide strong evidence that they are representing short sequences of states. Between-subject measures of this implicit sequential inference strategy had a neurobiological underpinning and correlated with grey matter density in prefrontal and parietal cortex, as well as the hippocampus. Our findings provide, to our knowledge, the first evidence for sequential inference in human cognition, and by exploiting between-subject variation in this measure we provide pointers to its neuronal substrates.

## Author summary

When studying human cognition, it is often assumed that agents form and update beliefs only about the current state of the world, an approach known as Bayesian filtering. However, in many situations there are advantages to making inferences about the most likely sequence of states that have occurred, which involves simultaneously updating beliefs about the present and the past, based on incoming information. Currently, very little is known about whether humans adopt such sequential inference strategies, and if they do, about the neuronal mechanisms involved. We addressed this by applying computational modelling to data collected during a probabilistic reversal task. At a group level, subjects’ behaviour showed clear evidence of sequential inference, and between-subject differences in the strategies adopted were reflected in variations in brain structure in the prefrontal and parietal cortices, as well as the hippocampus. Our results provide new insight into the strategies employed in human cognition, as well as the neuronal substrates of sequential inference.

## Introduction

Model-based approaches to cognition posit that agents continually perform online inference about the current state of the world, based on incoming sensory information. Typically, these approaches assume that the agent optimises beliefs about the current state of the world, referred to as Bayesian filtering. However, in many situations (for example, when parsing language) it is more appropriate to optimise beliefs about a sequence of states. Since the joint probability of a sequence of states is not, in general, the same as the (product of marginal) probabilities of the individual states considered individually, this leads to two alternative definitions of optimality: optimality of inference about individual states, and optimality of inference about sequences of states. These diverging goals are captured in the sum-product and max-sum algorithms for exact inference [1]).

**Fig 1:**
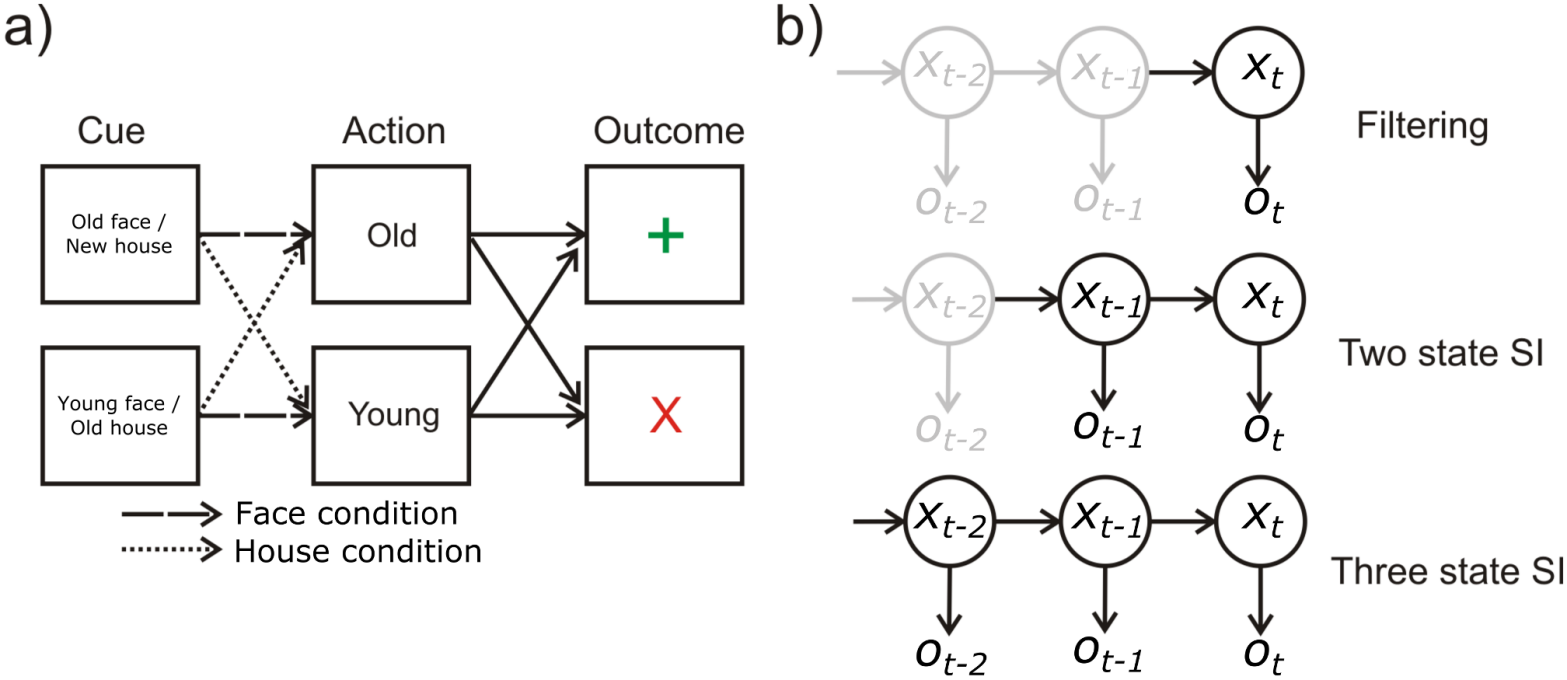
(a) Probabilistic reversal task. On each trial, subjects were presented with one of two compound stimuli. These consisted of overlaid images of either an old face and young house (top) or young face and old house (bottom). Subjects were instructed to respond ‘old’ or ‘young’ depending on whether they were in the face or house condition, and that this condition would switch periodically. They were then given feedback based on their choices, with a reliability of 0.85 (in other words, correct decisions resulted in positive feedback on 85% of occasions) (b) Cartoon illustrating sequential inference. An agent implementing pure filtering performs inferences about the current state (*x*_*t*_) based on fixed beliefs about the preceding state (*x*_*t-1*_), together with a current observation (*o*_*t*_). In *n* step sequential inference, the agent simultaneously optimises beliefs about both current and past states (up to a depth *n*). This corresponds to performing inference over a sequence of *n+1* states, rather than only performing inference over the current state. Under conditions where states are only partially observable (where there is no one-to-one mapping between states and observations), and where there are significant causal dependencies between states, this has the potential to significantly improve model performance.

In the context of offline data analysis, it is common to calculate the most likely sequence of states across an entire data set using the Viterbi algorithm, an application of the max-sum approach [2]. However, for embodied agents performing online inference, these schemes are not an option because future outcomes are unobservable, while inference over long sequences becomes computationally intractable. One possibility is that agents instead perform windowed sequential inference, in other words they perform inference about short sequences of states stretching back into the past (Fig 1b). Compared with Bayesian filtering this entails relatively minor increases in computational cost and represents a plausible hypothesis regarding implementation of actual cognitive processes in human subjects.

The advantage of representing ‘a short history’ of states is that the most likely (posterior) distribution over states becomes a more accurate approximation to the true posterior, where the true posterior entails conditional dependencies between states and different times. For example, if I am hungry at 11 AM I am more likely to be taking lunch at 1 PM. One cannot represent this belief in terms of the statements “I was hungry at 11 AM and I was lunching at 1 PM”. Although a Bayesian filter would correctly infer a higher probability of having lunch at 1 PM, given I was hungry earlier, its posterior belief about the current state having lunch has nothing to say about preceding hunger. Sequential inference becomes even more prescient when we consider Non-Markovian processes. Bayesian filters assume that states of the world evolve in a Markovian fashion, such that the preceding state completely specifies the next state in a probabilistic sense. However, in real-world situations (e.g. language), this Markovian assumption is often violated. For example, the semantic violation at the end of a sentence depends on the sequence of preceding words, not just the penultimate word. In this sense, inferring sequences with deep temporal structure necessarily requires the brain to perform some form of sequential inference. The importance of considering information contained at the level of entire sequences has been elegantly demonstrated in work on speech recognition using machine learning, where models that learn at the level of entire sequences consistently outperform those that learn at the level of individual frames (short epochs) [3–6].

Establishing whether human subjects perform sequential inference has important implications both for models of cognition and their neurobiological underpinnings. In particular, sequential inference requires that agents explicitly represent and update beliefs about states in the past, as well as the present. This mandates that neuronal circuits should also represent past states, and suggests that brain areas involved in processes such as maintaining and manipulating information about the past, for example prefrontal cortex and hippocampus (i.e., as organs of succession), might play a crucial role.

**Fig 2:**
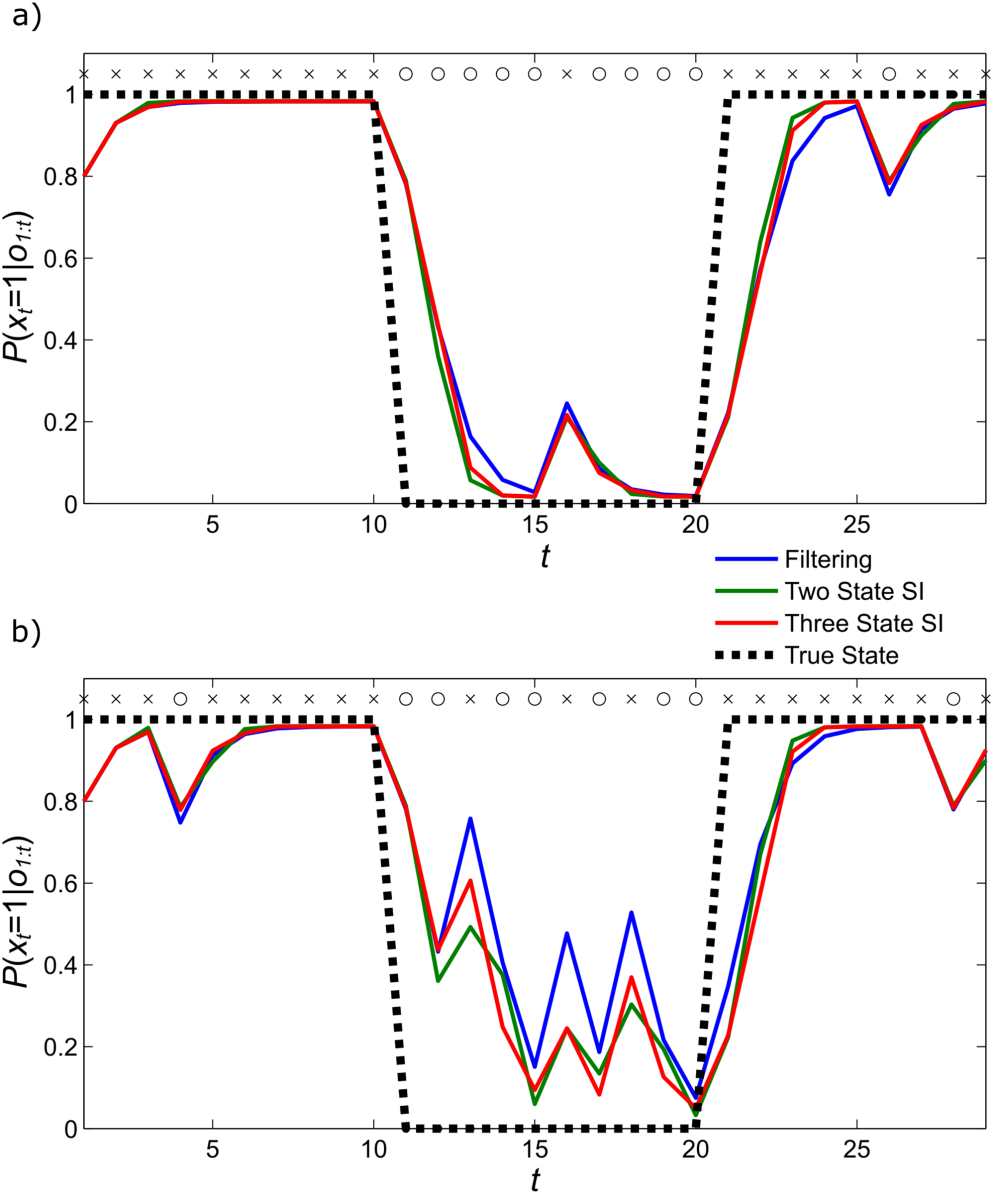
Simulated beliefs for agents employing different sequential inference strategies in performing inference on two different series of observations (Fig. 2a and Fig 2b). Observations on each trial are indicated by crosses and circles, while the ‘true’ state (i.e., task relevant category) is indicated by the dotted line. Reversals occur when the true state changes value (from zero to one, or vice versa) Posterior probabilities for state one obtaining (in other words, the agents’ beliefs at the end of each trial) are shown for the filtering model (S1) in blue, for the two state sequential inference model (S2) in green and the three state sequential inference model (S3) in red. In this case, sequential inference leads agents to track reversals more quickly. This is indicated by the red and green lines shifting towards the true value more quickly after reversals. Sequential inference also reduces the effects of misleading observations, as indicated by smaller changes in belief in response to unexpected stimuli that do not signal reversals (Simulations were performed with parameter values r = 0.05 and v = 0.8, see *Hidden Markov Model* for description).

Importantly, filtering and sequential inference strategies make quantitatively distinguishable predictions about behaviour on appropriate tasks (Fig 2), permitting us to compare the evidence for these strategies through behavioural modelling Thus, to test our hypothesis that human subjects perform sequential inference, we developed a simple computational scheme to implement sequential inference within the context of a probabilistic reversal task (Fig 1a). On each trial of this task, subjects were presented with superimposed images of either a young face and an old house, or an old face and a modern house. Subjects were asked to respond (either ‘old’ or ‘young’) and were given probabilistic feedback based on whether the response was correct. The correct response was determined by the (unknown) task relevant category (either ‘face’ or ‘house’), which the subjects had to infer, based on feedback. Crucially, the task relevant category or condition reversed periodically requiring subjects to track switches in the category condition; in order to maintain performance (see Methods for more details).

We applied sequential inference models to behavioural data from 43 younger and 36 older adults (Fig 1). We hypothesised that behaviour would show evidence of sequential inference, and as this depends on working memory type resources it might also show an age-related decline. To identify potential neuronal substrates of sequential inference, we analysed structural magnetic resonance imaging (MRI) scans to test for the correlates of sequential inference in terms of grey matter density using voxel-based morphometry (VBM) [7]. We hypothesised that an ability to exploit sequential inference would be positively correlated with grey matter density in brain regions involved in constructing models of the environment and maintaining on-line information, specifically the anterior or dorsolateral prefrontal cortex and hippocampus.

## Results

### Model-free behavioural analysis

Overall, subjects made correct choices (defined in terms of the actual task contingencies) on 74.5% of trials (SEM: 0.795). As expected, younger adults made more correct choices (mean: 77.9% SEM: 0.765) than older subjects (mean: 70.4% SEM: 1.172). This difference was statistically significant (*p* < 0.0001, Wilcoxon rank sum test), suggesting, in line with a previous literature (see [8] for review), younger adults were considerably better at decision making on this probabilistic reversal learning task.

### Preliminary model comparison

To confirm the appropriateness of the Bayesian modelling approach (see Methods), we performed a preliminary model comparison where we compared the performance of simple Bayesian filtering (*S1*) with three models based on *Q*-learning. These consisted of a simple model in which each action value was updated independently (*Q1*), a model in which all values were simultaneously updated (*Q2*), and a model in which all values were simultaneously updated but with separate learning rates for positive and negative feedback (*Q3*). In keeping with previous findings [9–11], random effects model comparison favoured *S1* in both younger (exceedance probability > 0.99) and older (exceedance probability = 0.94) groups (Table A in S1 Text). This suggests that the behaviour of the subjects do indeed takes into account the structure of the task, in particular the fact that it involves abrupt shifts (reversals), rather than gradual but continuous changes.

### Sequential inference analysis

**Fig 3:**
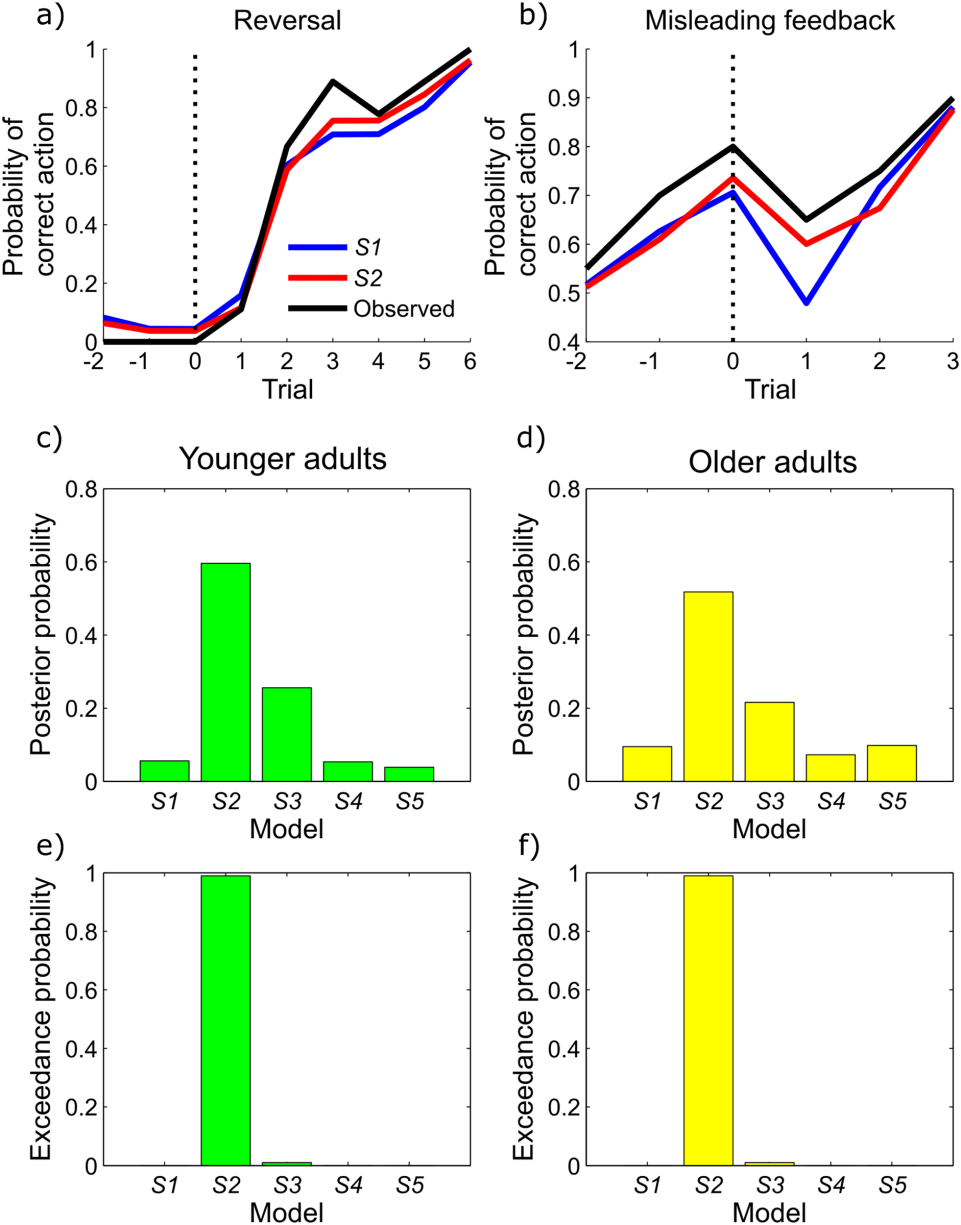
(a,b) Mean choice data and fitted model predictions from a single illustrative (younger) subject in whom the two state sequential inference model (*S2*, red) was strongly favoured over filtering (*S1*, blue). 3a shows model predictions and observed choices averaged across reversals. 3b shows predicted and observed behaviour averaged across misleading outcomes (situations where the contingencies had not reversed, but improbable feedback was observed suggesting that they might have). The two state sequential inference model provides a better fit to the observed choice data (black) than the filtering model because it permits a combination of more rapid switching (3a) and damped responses to misleading observations (3b), precisely in keeping with our simulation results (Fig 2). The *y* axis indicates the probability of taking the action that is correct after the reversal in 3a, and the action that is correct throughout the whole time window in 3b. (c-f): Model comparison for behavioural data in younger (3c,e, green) and older (3d,f, yellow) subjects. In both age groups, two-state sequential inference (S2) was strongly favoured as the best model, as evidenced by its very high exceedance probabilities (e,f). However, inspection of the posterior probabilities suggested a diversity of strategies across subjects; with the use of three state sequential inference (S3) in particular being strongly supported by the data. (This figure illustrates the results presented in Table 1).

To test whether subjects performed sequential inference, we compared the behavioural fits of pure filtering model (*S1*) with those in which subjects performed inference over sequences of lengths two to five (*S2-5*). Random effects model selection provided evidence that both younger and older adults performed inference over sequences of length two or more (Table 1, Fig 3). The favoured model in both groups (with an exceedance probability of 0.99 or greater) was *S2*, in which agents perform joint inference over the current state and the preceding state, but with evidence of significant variability in sequence length between subjects.

**Table 1:** Behavioural analysis of sequential inference strategies. In both younger and older adults, random effects model comparison results strongly favour *S2*, a model in which agents perform joint inference over the current and immediately preceding states. Examination of the posterior probabilities, however, suggests that a significant proportion of subjects in both groups performed inference over sequences of length three or more (model comparison results are illustrated in Fig 3c–3f).

Model (sequence length)	Summed BIC	BIC compared to worst model	Posterior probability	Exceedance probability	Mean Pseudo R^2^
***Younger adults (n = 43)***					
***S1***	-3901.1	0.9	0.056	<0.001	0.613
***S2***	-3809.7	92.2	0.596	0.996	0.625
***S3***	-3822.6	79.3	0.256	0.004	0.624
***S4***	-3887.4	14.6	0.053	<0.001	0.614
***S5***	-3902.0	0	0.039	<0.001	0.612
***Older adults (n = 36)***					
***S1***	-4554.5	0	0.095	<0.001	0.376
***S2***	-4528.5	26	0.518	0.990	0.380
***S3***	-4537.7	16.8	0.216	0.010	0.379
***S4***	-4547.1	7.4	0.073	<0.001	0.377
***S5***	-4554.4	0.1	0.098	<0.001	0.376

Bayesian parameter averaging over the model space showed that younger adults had significantly higher values for both *r* and *v* than older adults (Younger: *r* = 0.95, *v* = 0.86. Older: *r* = 0.89, *v* = 0.72. Both *p* < 0.001, Wilcoxon rank sum test). This corresponds to beliefs that the environment is more stable, meaning a reversal is less likely to occur, and that feedback was more informative. In younger adults, these beliefs also reflect the true contingencies in the task. It thus appears that both age groups perform inference over a similar sequence length, while the age group effect in choice accuracy might be better captured by the certainty of the prediction at each given state, which we investigate in more depth elsewhere [12]. Note that since we fixed the precision parameter in our model fitting, due to the redundancy between model parameters [9,13], some of these group differences may in fact reflect the influence of changes in precision *γ*, which governs the stochasticity of action selection (Eq 4). Given that our main model comparison suggested similar distributions of sequential inference strategies across age groups, we pooled across subjects, including age group and model fit quality as indexed by the mean BIC score over the model space for each subject as confounding regressors in subsequent VBM analyses. Measures of model fit such as the BIC and pseudo-R^2^ indicated that all models explained less data variance in older adults (Table 1). This is unsurprising given the differences in parameter estimates described above, which indicate that older subjects’ behaviour is more stochastic and harder to predict.

### Structural correlates of sequential inference

We next performed voxel-based morphometry [7] to explore how regional variations in grey matter density are related to interindividual differences in sequential inference. Our motivation here rested on well described relationships between regional grey matter density and cognitive function across a variety of domains [14]. This led us to hypothesise that grey matter density would provide a marker that would allow us to identify regions that play a key role in implementing sequential inference. To characterise between-subject variability in cognitive strategy, we used two key, and complementary measures. First, as a measure of how strongly sequential inference (as opposed to filtering) influenced subjects’ behaviour, we defined a measure *ΔLL* as the difference in the accuracy with which the best sequential inference model and the filtering model predicted behaviour as quantified by the difference in log likelihood. Second, we defined a measure *L* which indexes the length of sequence considered by each subject based on the winning model for that subject. These were entered into a multiple regression model, along with control regressors encoding age group, gender, total intracranial volume, individual subject parameter estimates (*r* and *v*), and the mean BIC score across the model space for each subject. Note there was no correlation between estimates of *L* and *ΔLL* themselves (*R* = 0.020, *p* = 0.861)).

**Fig 4:**
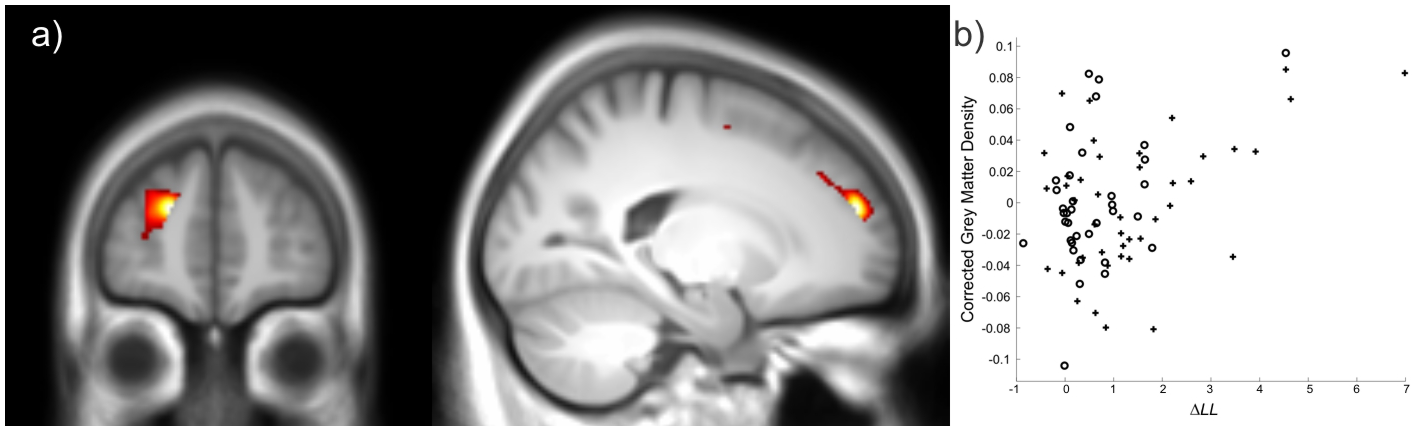
Voxel-based morphometry results for the *ΔLL* regressor encoding how strongly individual subjects’ behaviour showed evidence for sequential inference. 4a) *ΔLL* was positively correlated with grey matter density in the left anterior prefrontal cortex. (Image thresholded at *p* < 0.005, cluster size *k* > 10, uncorrected for display purposes. Slices depicted are at y = 53 (left) and x = -20 (right))4b) Scatter plot showing that both younger (crosses) and older (circles) subjects showed a similar positive relationship between *ΔLL* and grey matter density. (Grey matter density extracted from the group level peak [–20 53 20], and corrected for all other regressors in the design matrix).

Across subjects, *ΔLL* showed a positive association with grey matter density in the left anterior prefrontal cortex (putatively Brodmann area 10), a relationship significant at the whole brain level (peak voxel coordinates [–20 53 20], *t*_*70*_ = 5.08, *p* = 0.017 FWE-corrected for whole brain) (Fig 4, Table B in S1 Text). This suggests that this region may play a key role in determining a propensity to sequential inference, consistent with a dependence of sequential inference on higher-level cognitive functions, such as working memory maintenance and metacognitive functions, widely believed to be subserved by the anterior prefrontal cortex [15–18].

**Fig 5:**
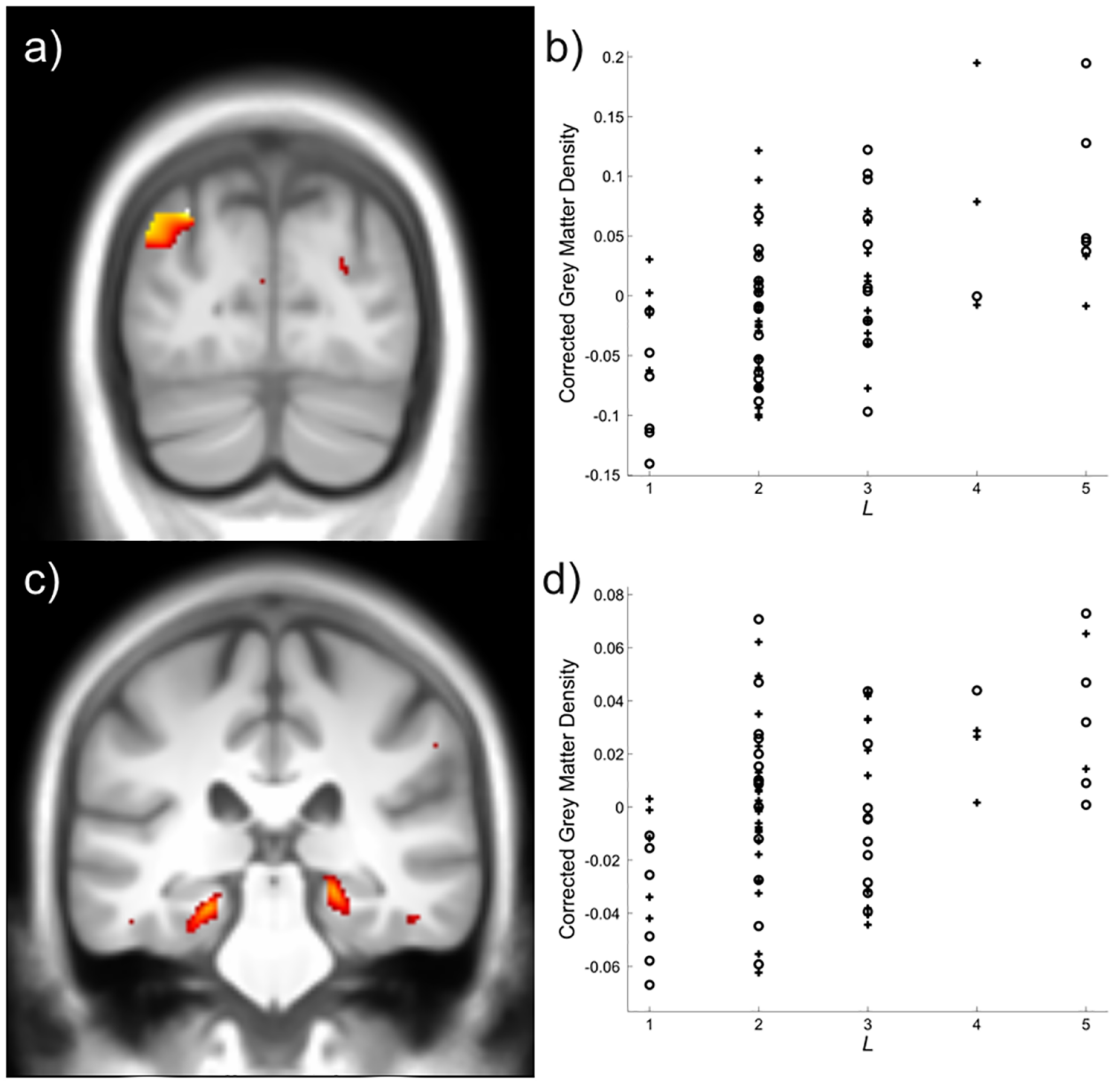
Voxel-based morphometry results for the *L* regressor encoding the sequence length individual subjects inferred over. 5a,c) *L* was positively correlated with grey matter density in the left posterior parietal cortex (a), and bilateral hippocampus (c). Images thresholded at *p* < 0.005 uncorrected, cluster size *k* > 10, for display purposes. Slices depicted are at y = -80 (a) and y = -30 (c)5b,d) Scatter plots showing that both younger (crosses) and older (circles) subjects showed a similar positive relationship between *L* and grey matter density in both the posterior parietal cortex (b) and bilateral hippocampus (d) Grey matter density extracted from the group level peaks (posterior parietal cortex: [–29–80 39], hippocampus: [–23–30–17] and [21–30–12] collapsed across hemispheres), and corrected for all other regressors in the design matrix.

Between-subject differences in the sequential length regressor *L* were correlated with grey matter density in the left posterior parietal cortex ([-29–80 39], *t*_*70*_ = 4.84, *p* = 0.039 FWE-corrected for whole brain) (Fig 5, Table C in S1 Text). Regions of the posterior parietal cortex are widely implicated in memory recollection [19–22], and maintenance in working memory [23,24]. In addition, based on its key role in encoding sequences [25–28], we performed a region of interest analysis in the hippocampus, using coordinates derived from previous work on recollecting temporal sequences [27]. Grey matter density in bilateral hippocampus positively correlated with *L* (Fig 5, Table C in S1 Text) (Left: [–23–30–17], *t*_*70*_ = 3.75, *p* < 0.007 FWE-corrected for hippocampus ROI volume. Right: [21–30–12], *t*_*70*_ = 3.68, *p* < 0.008 FWE-corrected for hippocampus ROI). This provides clear evidence that the hippocampus plays a role in supporting sequential inference strategies.

Finally, to assess whether grey matter density in the anterior prefrontal cortex also showed a relationship with sequence length, we performed an additional region of interest analysis using the prefrontal cluster identified in our *ΔLL* analysis (thresholded at *p* < 0.001 uncorrected). Importantly, because these regressors are entered into a single multiple regression analysis, we control for the effect of the log likelihood regressor. This analysis showed a clear positive relationship between grey matter density in anterior prefrontal cortex and sequence length ([–21 56 21], *t*_*70*_ = 2.76, *p* = 0.031 FWE-corrected for ROI), further implicating this region in implementing sequential inference. There was no relationship with *ΔLL* in the angular gyrus cluster or hippocampus ROIs.

*Post hoc* analyses suggested that the relationship between our measures of interest and grey matter density in these regions were similar across age groups, suggesting that the neuronal substrates of sequential inference do not alter over the course of healthy ageing (see S1 Text). No areas showed a significant negative relationship with ΔLL or L, and no significant effects obtained for any of the other regressors (including the parameter estimates r and v), other than age and total intracranial volume, which correlated with widespread changes (decreases for age, increases for total intracranial volume) in grey matter density.

### Sequential inference and other cognitive measures

To explore possible relationships among our measures of sequential inference and performance on other cognitive tasks, we used a letter n-back task to measure working memory and Raven’s matrices to test non-verbal reasoning [29]. The n-back task is described in more detail in [30]. Briefly, subjects were presented with a series of letters and asked to indicate with a button press for each letter whether it is the same as the letter 1, 2 or 3 letters back. To test for inter-individual differences in fluid intelligence, an abbreviated version of Ravens matrices was used (odd matrices from levels C, D and E, resulting in 18 matrices). Subjects were given 20 minutes to complete the matrices. We calculated partial correlations, controlling for the effects of age group and gender. Working memory performance, as assessed by (Hits—False alarms), averaged across one-back, two-back and three-back conditions, showed a positive correlation with *L* that showed trend significance (*R* = 0.218, *p* = 0.057). No clear relationship was found with *ΔLL* (*R* = -0.041, *p* = 0.724). No clear relationships were found between Raven’s matrices scores and either *L* (*R* = 0.101, *p* = 0.408) or *ΔLL* (*R* = 0.043, *p* = 0.727). Note that four younger and three older subjects were excluded from the Raven’s matrices analyses, and five younger and five older subjects from the working memory analyses, because they did not complete the relevant tasks. No structural correlates of working memory performance or Raven’s matrices scores were found in additional VBM analyses.

## Discussion

Sequential inference represents a significantly different approach to solving inference problems from Bayesian filtering. In this framework agents are required to infer multiple states, and their dependencies, simultaneously rather than just infer the current state. This requires a capability to store information about previous observations, as well as maintain and update information about joint distributions. Here, we provide evidence that both younger and older adults make use of such strategies, inferring the joint probability of sequences of states rather than inferring the current state in isolation and use this inference to inform their choice behaviour. Our findings represent an important departure from existing models of human performance on inference tasks, and provides new insights into the cognitive and computational strategies used by human subjects for coping with a changing and uncertain environment.

Performing inference over sequences of states allows an agent to consider processes that violate the Markov assumption, under which the current state is determined only by its immediate precursor. This allows agents to deal with problems that have deep temporal structure, as is common in many real-world situations such as language processing. Indeed in models used for speech recognition, training at the level of sequences has been shown to produce considerable benefits over training at the level of individual frames [3–6], highlighting the benefits of considering deeper structure. To see the limitations imposed by the Markov assumption, consider the task of predicting whether it will rain today or not. The weather yesterday undoubtedly provides some information about this, but there is also extra information contained in observations taken on previous days, since the weather is determined by processes at timescales from the relatively rapid (individual rain clouds) to the very slow (seasonal fluctuations, El Niño). Sequential inference thus equips agents with greater flexibility, which is likely to be important for understanding both normative strategies and human behaviour. Moving beyond Bayesian filtering also has major implications for learning (the so-called ‘dual estimation problem’ [31]), which we will consider in future work.

In addition, using a model-based analysis we were able to relate between-subject differences in sequential inference performance to grey matter density in left anterior prefrontal and left posterior parietal cortex, as well as bilateral hippocampus. This is consistent with the established role of these regions in supporting the computations necessary for performing inference over extended sequences of states, given an established relationship between morphometric features of a region and its level of functional engagement [14].

A striking (very significant) finding in our voxel-based morphometry was the link between left anterior prefrontal cortex, encompassing Brodmann area 10, and the strength of evidence for sequential inference. This region is believed to play a role in higher order executive cognitive processes [16,32,33],memory retrieval [34] and the manipulation of internally generated information [35]. It is also implicated in metacognition [18,36]. In particular, it has been proposed that the anterior prefrontal cortex plays a key role in ‘cognitive branching’, where multiple cognitive processes or options for action need to be maintained simultaneously and integrated together [15], processes with similarities to the type of updating subjects perform based on our model. Additionally, a similar region of prefrontal cortex is implicated in retrospective (‘metacognitive’) judgements about decision confidence, albeit with evidence to suggest this is lateralised to the right hemisphere [18,36]. Unfortunately, it is not possible—based on our data—to draw fine-grained inferences about the precise cognitive processes that this region supports during sequential inference, something we will address in future computational and experimental work.

We observed that interindividual differences in sequence length, likely to be associated with increasing mnemonic demands, positively correlated with grey matter density in a region of the left posterior parietal cortex and bilateral hippocampus. Areas of the posterior parietal cortex are activated in neuroimaging studies of memory recall, but the precise functional role of this activity remains the subject of much on-going debate [19–22]. Additionally, the parietal cortex has been implicated in maintaining information in working memory [23,24]. Either or both processes could be involved here and drive the observed relationship. Alternatively, the relationship between sequential inference and grey matter density in this area might reflect a role in some other cognitive operation that underpins sequential inference, perhaps in concert with the hippocampus.

The association between the length of sequence considered by a subject and grey matter density in the hippocampus is consistent with its known role in encoding, maintaining and recalling sequences [25–28]. Our data suggest a new role for such retrospective representations, namely in supporting sequential inference. More speculatively, it is tempting to link the hippocampus’ putative role to the internally generated forward and backward sweeps through state space it is believed to support [37–39]. Such sweeps are typically considered in the context of planning and navigation, but they are also ideally suited for use in inference and learning more generally [37,40].

In this study, we considered behaviour and brain data collected from both younger and older adults. Although there were clear differences in task performance (and, correspondingly, in model parameter estimates) between groups, similar distributions of sequential inference strategies were observed in both groups, suggesting that these differences were unlikely to be explicable in terms of the deployment of different cognitive models. Closely related to this, a *post hoc* analysis of our structural data suggested similar relationships between our measures of sequential inference and regional grey matter density in both groups. Taken together, these results suggest that (at least within the restrictions of our data), the deployment of sequential inference is largely conserved over the course of healthy ageing.

The results have implications for future studies using reversal paradigms with probabilistic outcomes, particularly in humans [11,41–44]. Our results suggest that most subjects perform sequential inference. For many purposes (for example when deriving regressors for functional neuroimaging), the differences between filtering and sequential inference model predictions are likely to be unimportant. However, they may be relevant for understanding between-subject variability, for example in patient groups who show reversal learning impairments [9,45–49].

A number of studies have suggested that medial prefrontal and orbitofrontal regions are important in reversal learning, linking stimuli and outcomes [42,50–52]. This does not conflict with our findings, which instead predict that lesions of the anterior prefrontal cortex, posterior parietal cortex or hippocampus might result in subtler behavioural abnormalities, reflecting a decrease in sequential inference performance relying on such links between stimuli and outcomes. More generally, we propose that sequential inference modelling is a plausible approach for explaining behaviour across a wide variety of paradigms that engender decision uncertainty, for example in reward learning tasks with slowly drifting outcome probabilities [53,54]. We anticipate this will be a productive area for future research.

Sequential inference is a potential explanation for postdictive phenomena in perception [55–57]. In such phenomena, for example the flash lag illusion [56], the perception of a stimulus is influenced by stimuli presented after it. This retrospective influence could be explained if perception depends upon inference about the joint probability of events within some finite temporal window, since this will lead to subjects perceiving the most likely sequence of events, and hence allow later observations to inform percepts about earlier time points (for a related suggestion see [58]).

Our study provides the first behavioural evidence that human subjects perform simultaneous inference over both current and past states, a process likely to be important for adaptive behaviour. The link to regional cortical morphometry is of particular interest as the processes that support sequential inference include many that are supported by these regions. The findings also naturally suggest future lines of enquiry, including exploring the capacity for sequential inference in supporting adaptive behaviour in other cognitive contexts engendering uncertainty as well as in patient groups who manifest abnormal performance on inference tasks.

## Methods

### Participants

Our sample comprised 79 participants encompassing two age groups: 43 younger adults (18 male, mean age = 26.4 years, range = 20–33 years) and 36 older adults (20 male, mean age = 66.4 years, range = 60–73 years). Data from five younger adults and 2 older adults were excluded due to technical problems during data acquisition. The educational level of the participants was comparatively high: 43% of the younger adults had attended the Gymnasium, a University high school preparatory track and 51% were currently enrolled at University. Most of the older adults held academic high school diploma (54%) or vocational school diploma (39%). All subjects were right-handed (Oldfield Questionnaire: LQ > 80; [59]). None of the participants reported cardiovascular pathology, psychotropic medication usage, history of neurological or psychiatric episodes or substance abuse.

The study was approved by the local ethics committee of the Charité, University Medicine Berlin, and written consent was obtained from each participant prior to participation. Participants were paid 10 Euros per hour of the experiment.

In a separate test session, the Digit Symbol Substitution test (DSS) [60] and a modified version of the Spot-a-Word test [61] were assessed as markers of fluid and crystallized intelligence, respectively. As expected, DSS performance decreased from early to late adulthood (*t* = -3.99, *p* < .01), whereas performance on the Spot-a-Word showed a trend for an increase with age (*Z* = 1.72, *p* = .08). The observed dissociation between lifespan age gradients of these two tests in our sample is consistent with established empirical evidence on the development of these two facets of intelligence (cf. [62]) and indicates that our sample falls within a representative range of age-comparative cognitive testing.

### Experimental task

Subjects performed a probabilistic reversal task across four sessions in the scanner during fMRI, giving a total of 128 trials. We will report the fMRI data in future papers. Here, our focus is on intersubject behavioural variability in the depth or prevalence of sequential inference and its anatomical correlates as measured with structural MRI.

The probabilistic reversal task involved focusing on either a face or house attribute of translucent greyscale images comprising faces and houses (Fig 1). Face and house stimuli were divided into either young and old faces or houses of modern and old styles respectively. Subjects had to learn whether to focus on the face or house dimension of the superimposed stimuli. On each trial, subjects were tasked categorise (the face or house) as young (modern) or old, with a left and right button press. A young face was always paired with an old house and vice versa. In this manner, feedback as to a correct young/old categorization was informative with respect to task set (with respect to faces or houses). Feedback was probabilistic, with 85% reliability. If the proportion of gains exceeded 80% on the most recent 10 trials, a reversal of the task relevant category (face or house) was implemented within the next 1–3 trials. Subjects were informed about the possibility of this change but not its actual timing or performance dependency. Subjects were instructed to try to obtain as many rewards as possible. This was achieved by inferring the task relevant (i.e. more frequently rewarded) category as well as switches therein. Subjects were familiarised with the task in a practice sessions that included at least one reversal. There were 4 runs in total, amounting to a total of 128 trials for the probabilistic reversal task. Within a trial, each stimulus was presented for 2 seconds, followed by a variable interval of 1–7 seconds (mean 1.25 seconds), during which a fixation cross was shown. This was followed by a feedback stimulus presented for 1 second and a variable interval of 2–8 seconds (mean 3.25 seconds) with a fixation cross (Fig 1).

Face stimuli were taken from the FACES database [63], house stimuli were selected from an internet search. Individual face and house stimuli were adjusted in brightness, overlapping face and house stimuli were adjusted separately for each specific stimulus pairing to ensure equal subjective saliency of face and house stimuli [64]. The gender of the face stimuli was balanced within tasks, as was the number of young (modern) and old stimuli. Before the task, subjects were familiarized on a different stimulus subset with the type of face and house stimuli that were used during the experiment, as well as with the categorization of individual or overlapping stimuli into young (modern) or old face and houses. Each stimulus was presented for 2 seconds, followed by a variable interval of 1–7 seconds (mean 1.25 seconds), during which a fixation cross was shown. This was followed by a feedback stimulus presented for 1 second and a variable interval of 2–8 seconds (mean 3.25 seconds) with a fixation cross.

### Behavioural covariate measures

To assess the contribution of cognitive function to differences in sequential inference, subjects performed a working memory (*n*-back) task, as well as the Raven’s matrices test of nonverbal reasoning. Performance measures on these tasks were used as additional covariates to analyse behaviour and brain structure.

### Hidden Markov Model

To model subjective inference, we used a simple Hidden Markov Model (HMM) in line with the previous literature [9–11]. Here, agents infer the hidden state *x*_*t*_ corresponding to the current task condition or context; in other words, the task relevant category, face or house. (We arbitrarily define *x*_*t*_ = 1 as the ‘face’ condition and *x*_*t*_ = 2 as the ‘house’ condition). The outcome of each trial is indicated by *o*_*t*_ (*o*_*t*_ = 1 represents positive feedback, *o*_*t*_ = 2 represents negative feedback). The visual cue presented on each trial is represented by *y*_*t*_, where *y*_*t*_ = 1 corresponds to the old face / modern house pair, and *y*_*t*_ = 2 corresponds to the young face / old house pair. Actions selected by the subject are encoded as *a*_*t*_ = 1 for an ‘old’ response and *a*_*t*_ = 2 for the ‘young’ / ‘modern’ response. The parameter *r* encodes the probability of a reversal between trials, and *v* encodes the cue validity (the probability of receiving positive feedback given that the agent has made the correct decision, and negative feedback if not). Thus, the generative model considered by the agent takes the form:

$$P(x_{t}\;|\;o_{t},a_{t},y_{t},x_{t-1},r,v)=\frac{P(o_{t\;}|x_{t},a_{t},y_{t},v)P(x_{t}|x_{t-1},r)}{P(o_{t})}$$ (1)

$$\begin{array}{l} P\left(x_{t}=i|x_{t-1}=j,r\right)=E_{ij}\\ E=\left[\begin{array}{cc} 1-r & r\\ r & 1-r \end{array}\right] \end{array}$$ (2)

$$\begin{array}{l} P\left(o_{t}=1|x_{t}=i,a_{t}=j,y_{t}=k,v\right)=A_{ijk}\\ A_{1**}=\left[\begin{array}{cc} v & 1-v\\ 1-v & v \end{array}\right]\\ A_{2**}=\left[\begin{array}{cc} 1-v & v\\ v & 1-v \end{array}\right] \end{array}$$ (3)

Action selection followed a softmax decision rule [54] based on the agent’s current beliefs such that:

$$\begin{array}{l} P\left(a_{t}=i|y_{t}=1,\gamma \right)=\frac{e^{\gamma P\left(x_{t}=i|o_{1:t-1}\right)}}{\sum_{j=1,2}e^{\gamma P\left(x_{t}=j|o_{1:t-1}\right)}}\\ P\left(a_{t}=i|y_{t}=2,\gamma \right)=1-P\left(a_{t}=i|y_{t}=1,\gamma \right) \end{array}$$ (4)

Where *γ* is the precision parameter that governs the stochasticity of choice. For completeness, we also fitted models that additionally included a ‘perseverance’ parameter indexing a tendency to repeat previously performed actions [13], but these proved to be inferior during model comparison, and we do not report these results here.

### Sequential inference

Having defined the relationship between successive states, as well as between states and observations, we created five Bayesian update schemes: a pure filtering scheme, which updates beliefs about the current state (a sequence of length one), and sequential models that performed inference over the current state and between one and four preceding states. In other words, the joint distribution over sequences of two to five states. Omitting the dependence on parameters, actions and stimuli for the sake of clarity, for a sequential inference model of order *n*, the agent performs inference over:

$$P\left(x_{t-n+1},\ldots ,x_{t}|o_{t-n+1},\ldots ,o_{t},x_{t-n}\right)$$ (5)

Thus the agent infers the joint probability distribution over the sequence of states *x*_*t-n+1*_ to *x*_*t*_, conditioned only on the last state preceding the sequence *x*_*t-n*_ and the series of outcomes *o*_*t-n+1*_ to *o*_*t*_. This means that for a model of order one (a filtering model), agents perform inference only over the current state:

$$P\left(x_{t}|o_{t},x_{t-1}\right)=P\left(o_{t}|x_{t}\right)P\left(x_{t}|x_{t-1}\right)$$ (6)

While for a model of order two, agents perform inference over the joint probability of a two-state sequence:

$$P\left(x_{t},x_{t-1}|o_{t},o_{t-1},x_{t-2}\right)\propto P\left(o_{t}|x_{t}\right)P\left(x_{t}|x_{t-1}\right)P\left(o_{t-1}|x_{t-1}\right)P\left(x_{t-1}|x_{t-2}\right)$$ (7)

The crucial difference between Eqs 6 and 7 is that the posterior in the pure filtering scheme is only over the current state, whereas for the sequential scheme (Eq 7) the posterior representation is over the joint occurrence of the current state and preceding state. Exact probabilities for the current state can then be calculated by first normalising and then summing probabilities. Thus for a sequential model of order two:

$$P\left(x_{t}=i|o_{1:t}\right)=\sum_{j=1:2}P\left(x_{t}=i,x_{t-1}=j|o_{t},o_{t-1},x_{t-2}\right)$$ (8)

Exact inference on the joint distribution over states requires a number of sufficient statistics that grows exponentially with the number of time steps considered (for this model 2^*n*^ statistics are necessary). Since this rapidly becomes combinatorially intractable, we consider only short sequence lengths of 1 to 5 states (this choice was endorsed by our model comparison results, which favoured short sequences).

It is important to note that although sequential inference permits an agent to consider non-Markovian processes (in other words, processes with deep temporal structure), the generative models considered here are all Markovian. The dissociable behavioural predictions that we consider result from the fact that filtering and sequential inference strategies optimise different beliefs as described above. Exploring sequential inference in the context of problems with non-Markovian structure is an issue that we will consider in future work.

### Action value models

In addition, we performed a preliminary analysis demonstrating the applicability of the HMM to our data by fitting three different models based on variations of action value learning [65]. In the simplest, ‘single update’ model (*Q1*), the value of each state-action pair *Q*(*y*_*t*_,*a*_*t*_) is updated separately based on a fixed learning rate *φ* and the difference between the observed and the expected reward (the reward prediction error):

$$Q\left(y_{t},a_{t}\right)=Q\left(y_{t},a_{t}\right)+\varphi \left(o_{t}-Q\left(y_{t},a_{t}\right)\right)$$ (9)

Actions were again selected according to a softmax decision rule, such that:

$$P\left(a_{t}=i|y_{t},\gamma \right)=\frac{e^{\gamma Q\left(a=i,y_{t}\right)}}{\underset{j=1,2}{\sum }e^{\gamma Q\left(a=j,y_{t}\right)}}.$$ (10)

Since this model ignores the reciprocal nature of the values of state action pairs, we also fit a ‘quadruple update’ model (*Q2*), in which the values of all four state-action pairs were updated simultaneously. Finally, we fit a ‘quadruple asymmetric update’ model (*Q3*) which contained separate learning rates *α*_*g*_ and *α*_*l*_ for positive and negative feedback, allowing for potential asymmetries in how subjects responded to gains and losses.

### Model fitting and comparison

Maximum a posteriori (MAP) estimation was performed with largely uninformative priors. These were defined as:

$$\begin{array}{l} r\;∼\;Beta(1.1,1.1)\\ v\;∼\;Beta(1.1,1.1)\\ \text{ln(}\gamma \text{) }∼\;N(1,0.25)\\ \varphi \;∼\;Beta(1.1,1.1) \end{array}$$

where *Beta*(*α*, *β*) is the beta distribution with parameters *α* and *β*, and *N*(*μ*, *σ*^2^) is the normal distribution with mean *μ* and variance *σ*^2^. These priors essentially served to prevent the parameters taking extreme values, but similar results were obtained using simple maximum likelihood estimation. Fitting was carried out using the Nelder-Mead simplex method. We selected MAP estimation because it is simple and computationally efficient, and it also allowed us to fit parameters in intuitively meaningful spaces whilst making only minimal assumptions (in contrast, for example, to using a Laplace approximation). However, we acknowledge that more sophisticated approaches could have been adopted and these may be fruitfully adopted in future studies.

Because there is a strong degree of redundancy between the softmax precision (or inverse temperature) parameter *γ* and other model parameters [9,13], to mitigate against overfitting we used a fixed *γ* across all subjects. This was fixed separately for each model, using the group mean from an initial analysis, in which *γ* was a free parameter: pairwise model comparison between fixed *γ* and free *γ* versions of each type of model provided strong evidence in favour of the fixed model in every case.

Model comparison was based on the Bayesian Information Criterion (BIC), which is defined as

$$BIC=2\Sigma_{t=1:n}\text{ln}P(at|o_{1:t-1},\theta_{MAP})-k\text{ln}(n)$$ (11)

where *k* is the number of free parameters in the model and *n* is the number of data points. To compare models we employed a random-effects model comparison approach, which is robust to the effects of group heterogeneity and outliers [66]. Parameter values for inclusion in the VBM analysis were derived using Bayesian parameter averaging over the sequential inference and filtering models. Briefly, this involves taking a weighted average of the estimated parameters for each model, with single-subject weightings determined by the posterior probabilities of each model in that subject.

### Scanning and voxel-based morphometry

Whole-brain structural MRI data were obtained with a Siemens 3T Trio Magnetom using a T1-weighted MPRAGE sequence (TR, 1550 ms; TE, 2.34 ms; TI, 900 ms; FA, 9 degrees; voxel size, 1 × 1 × 1 mm; no gap; FOV, 244 ms). Structural scans were segmented, aligned and normalised to MNI space at a resolution of 1.5 mm isotropic using DARTEL in SPM12 [67], and smoothed with an 8mm kernel. Total intracranial volumes were calculated as the summed volume of the grey matter, white matter and CSF images derived using the SPM new segmentation functions [68]. VBM analysis was performed using the normalised grey matter images, masked at a threshold of 0.2.

To test for structural correlates of sequential inference we defined two key measures. First we defined a measure of how strongly sequential inference was manifest in each participant’s behavior. This measure, *ΔLL*, was defined as the difference in log likelihood scores between the best sequential inference and filtering models. We also defined a measure *L* which encoded the length of sequence under the best fitting model in each subject.

To test for the structural correlates of sequential inference using VBM, we defined a general linear model that contained these measures; as well as regressors encoding age group (as a binary regressor), gender, average model fit (as assessed by the mean BIC score), the parameter estimates *v* and *r* derived from Bayesian parameter averaging as described above, and total intracranial volume (in order to assess volume effects independent of overall interindividual differences changes in intracranial size [69]). All preprocessing and imaging analyses were performed using SPM12 (http://www.fil.ion.ucl.ac.uk/spm/).

Hippocampal regions of interest were defined based on the literature implicating the hippocampus in the processing of temporal sequences [27] using spheres of 10 mm radius centred at [+/-21–21–15] (MNI coordinates). To see whether grey matter density in areas sensitive to the propensity for sequential inference also reflected sequence length, we defined a region of interest in the anterior prefrontal cortex showing a correlation with *ΔLL* at a threshold of *p* < 0.001 uncorrected. Note that, under the null hypothesis, the change in log likelihood and depth of sequential inference are independent.

## Supporting information

S1 Text Post hoc analysis of structural data and age-related differences in structural inference. Tables giving preliminary model comparison and VBM results. Figures illustrating the effects of *ΔLL* and *L* on model fitting.
(DOCX)
