# Correction: Sequential inference as a mode of cognition and its correlates in fronto-parietal and hippocampal brain regions

**DOI:** 10.1371/journal.pcbi.1005908

**Published:** 2017-12-13

**Authors:** The PLOS Computational Biology Staff

The authors describe how the interpretation of the paper has changed as a result of this conceptual error below, though the analyses and results themselves are unaffected:

“During the course of extending on our prior work we became aware that our paper contains a conceptual error that alters the interpretation of the results we describe (though we add that the analyses and results themselves are unaffected).

In the paper we hypothesised that human subjects use a ‘sequential inference’ strategy, and provide evidence that our model, based on this hypothesis, captures behaviour on a probabilistic reversal task better than Bayesian filtering (where agents infer on the probability of individual states considered in isolation). On this basis we inferred that this constitutes evidence that humans adopt an alternative, but equally optimal, strategy to Bayesian filtering when performing the task.

Since publishing the paper, we have realised that this description has shortcomings. In fact, the sequential inference model that we use is suboptimal, for reasons we describe below. Moreover, a truly optimal sequential inference strategy makes behavioural predictions identical to Bayesian filtering on this task. Thus, our results do provide evidence that subjects infer over sequences of states stretching into the past as we originally claimed. We now also conclude that the data does not support a claim about normativity that we make in the paper.

The voxel-based morphometry results are, in themselves, unaffected by this error. However, interpreting the results is now less straightforward, due to the fact that optimal sequential inference produces identical predictions about behaviour as does filtering. In the case of the effects relating to the sequence length measure *L*, it is possible that subjects with *L* = 1 (11 out of 79 subjects) may be performing optimal sequential inference rather than filtering. Similarly, the measure *ΔLL* that indexes evidence for sequential inference can now be taken either to reflect evidence in favour of performing sequential inference as opposed to filtering (as we describe it in the paper), or evidence in favour of performing sequential inference suboptimally as opposed to optimally. Given the clear behavioural evidence that we found in favour of our sequential inference model, the interpretations given in the paper still seem reasonably compelling.

Notwithstanding the issue of interpretation we believe our work still makes a potentially valuable contribution. However, based upon our revisiting of the data we now consider that the findings need to be interpreted in a more nuanced fashion than described in the original publication.

## Mathematical explanation of error

To see why the model that we describe in the paper is suboptimal, consider a simple Hidden Markov Model (HMM) in which an agent seeks infers on a series of *T* time-varying hidden states *x*_1:*T*_ = {*x*_1_, …, *x*_*T*_} given a series of observations *o*_1:*T*_ = {*o*_1_, …, *o*_*T*_}. For clarity of exposition we drop the dependence on parameters *θ* and initial distribution *p*(*x*_0_), but these are nonetheless implied. We also define a sequence of states $\tilde{x}=\left\{x_{t-n+1},\ldots ,x_{t}\right\}$ and observations $\tilde{o}=\left\{o_{t-n+1},\ldots ,o_{t}\right\}$ where *n* is the length of the sequence considered by the agent, and *t* is the present time.

In Bayesian filtering, we recursively estimate beliefs about states at each time point *t* using the following equation:

$$p\left(x_{t}|o_{1:t}\right)=\sum_{x_{t-1}}p\left(x_{t}|o_{t}\right)p\left(x_{t}|x_{t-1}\right)p\left(x_{t-1}|o_{1:t-1}\right).$$ (C1)

In this paper, by contrast, we suggest that rather than inferring on individual states in isolation, agents might instead infer on the joint probability a sequence of states $p\left(\tilde{x}|o_{1:t}\right)$, and then calculate the marginal distributions over states at individual time points by summation:

$$\begin{array}{rr} p\left(x_{i}|o_{1:t}\right) & =\sum_{x_{j}}p\left(\tilde{x}|o_{1:t}\right)\text{,}\\ j & =\{j\in \mathbb{Z}:t-n+1\leq j\leq t\wedge j\neq i\}\text{.} \end{array}$$ (C2)

We then use this to derive a recursive algorithm where the agent infers on the joint probability of sequences of states using a sequence of observations $\tilde{o}$ and beliefs about the state immediately preceding the current sequence derived by marginalising the joint distribution at the previous timepoint. (In other words, the beliefs about that state generated during the previous round of sequential inference) Thus

$$p\left(\tilde{x}|o_{1:t}\right)\approx \sum_{x_{t-n}}p\left(\tilde{x}|\tilde{o}\right)p\left(x_{t-n+1}|x_{t-n}\right)p\left(x_{t-n}|o_{1:t-1}\right)$$ (C3)

This provides a compact algorithm that permits an agent to perform inference at the level of entire sequences, and update its beliefs about the past. However, as we have now realised, it is not strictly optimal. The HMM has the conditional independence property

$$p\left(o_{1:T}|x_{t-1},x_{t}\right)=p\left(o_{1:t-1}|x_{t-1}\right)p\left(o_{t:T}|x_{t}\right)$$ (C4)

(see (Bishop, 2006) for more details). This means that optimal inference over the sequence takes the form:

$$p\left(\tilde{x}|o_{1:t}\right)=\sum_{x_{t-n}}p\left(\tilde{x}|\tilde{o}\right)p\left(x_{t-n+1}|x_{t-n}\right)p\left(x_{t-n}|o_{1:t-n}\right)$$ (C5)

The difference here is in the final term, which specifies beliefs about the state *x*_*t*−*n*_ (the state immediately preceding the sequence being inferred on). When following an optimal strategy, agents should use *p*(*x*_*t*−*n*_ | *o*_1:*t*−*n*_), which is the estimate of this state derived from Bayesian filtering as given by (C1). This means that in our model observations at times early in the sequence are overweighted (effectively they are counted more than once), and leads to suboptimal inference (however, optimal sequential inference requires that an agent separately track filtered beliefs and those derived from sequential inference, which may be neurobiologically less plausible) Marginalising over the joint probability distribution generated by (C5) produces identical beliefs about the current state to those derived from Bayesian filtering.”
