# Supplementary material for: Sequential inference as a mode of cognition and its correlates in fronto-parietal and hippocampal brain regions
Source: PLoS Comput Biol. 2017 May 9;13(5):e1005418. doi: 10.1371/journal.pcbi.1005418 (PMC5441656; doi:10.1371/journal.pcbi.1005418)
Supplement: S1 Text — Tables giving preliminary model comparison and VBM results. Figures illustrating the effects of ΔLL and L on model fitting. (DOCX) [file pcbi.1005418.s001.docx]

Supplementary Information

Supplemental Results

*Post hoc analysis of structural data*

To explore whether grey matter density showed a similar relationship with our key measures across both age groups we extracted single subject grey matter density values (corrected for everything in the design matrix other than the variable of interest) from the peak locations described in our main analyses. We then performed Bayesian model comparison between general linear models containing either a single regressor encoding a constant effect size across age groups or two separate regressors encoding separate effect sizes. In all four regions, the single regressor model was strongly favoured (Table D).

*Age-related differences in sequential inference*

Although random effects model comparison strongly favoured a two-state sequential inference strategy in both younger and older adults, it remains possible that more subtle differences in sequential inference were manifest between groups. To test this, we compared *ΔLL* and *L* between groups using a multiple regression analysis that included gender and mean BIC scores as covariates of no interest (as in our VBM analysis). No significant relationship between age group and *ΔLL* or *L* were observed (*ΔLL*: *𝛽* = 0.36, *p* = 0.54. *L*: *𝛽* = 0.25, *p* = 0.41). Furthermore, similar results were obtained when using age as a continuous variable. This suggests, in keeping with our other findings, that there were no clear differences in sequential inference strategy between age groups.

Supplemental Tables

| **Model** | **Summed BIC** | **BIC compared to worst model** | **Posterior probability** | **Exceedance probability** | **Mean Pseudo R^2^** |
| --- | --- | --- | --- | --- | --- |
| ***Younger adults (n = 43)*** | | | | | |
| ***S1*** | -3901.1 | 1925.8 | 0.620 | 0.999 | 0.613 |
| ***Q1*** | -5826.9 | 0 | 0.022 | <0.001 | 0.308 |
| ***Q2*** | -4069.5 | 1757.3 | 0.128 | <0.001 | 0.567 |
| ***Q3*** | -4000.2 | 1826.7 | 0.230 | 0.001 | 0.602 |
| ***Older adults (n = 36)*** | | | | | |
| ***S1*** | -4554.5 | 729.1 | 0.525 | 0.943 | 0.376 |
| ***Q1*** | -5283.6 | 0 | 0.0835 | <0.001 | 0.208 |
| ***Q2*** | -4670.6 | 613.0 | 0.302 | 0.057 | 0.338 |
| ***Q3*** | -4650.9 | 632.7 | 0.0889 | <0.001 | 0.364 |

Table A: Preliminary model comparison results. The filtering model (*S1*) was strongly favoured over models based on action value learning, consistent with previous findings ^2,3,11^. The same pattern was observed in the older and younger groups considered independently. (*Q1*: single update model. *Q2*: quadruple update model. *Q3*: quadruple update model with separate learning rates for gains and losses)

| **Cluster** | | | **Peak** | | | | **MNI coordinates (mm)** | | |
| --- | --- | --- | --- | --- | --- | --- | --- | --- | --- |
| **p(FWE-corr)** | **equivk** | **p(unc)** | **p(FWE-corr)** | **T** | **equivZ** | **p(unc)** | **x** | **y** | **z** |
| 0.601 | 174 | 0.241 | 0.017 | 5.084 | 4.674 | 0.000 | -19.5 | 52.5 | 19.5 |
| 0.873 | 49 | 0.543 | 0.285 | 4.141 | 3.903 | 0.000 | -9.0 | -73.5 | 37.5 |
| 0.818 | 74 | 0.448 | 0.862 | 3.484 | 3.334 | 0.000 | -27.0 | -9.0 | 48.0 |
|  |  |  | 0.912 | 3.400 | 3.260 | 0.001 | -25.5 | -3.0 | 57.0 |

Table B: VBM results for regions showing a positive relationship with *ΔLL* (thresholded at *p* <.001 uncorrected, cluster size > 10 voxels).

| **Cluster** | | | **Peak** | | | | **MNI coordinates (mm)** | | |
| --- | --- | --- | --- | --- | --- | --- | --- | --- | --- |
| **p(FWE-corr)** | **equivk** | **p(unc)** | **p(FWE-corr)** | **T** | **equivZ** | **p(unc)** | **x** | **y** | **z** |
| 0.090 | 729 | 0.025 | 0.039 | 4.837 | 4.477 | 0.000 | -28.5 | -79.5 | 39.0 |
|  |  |  | 0.108 | 4.498 | 4.201 | 0.000 | -48.0 | -69.0 | 27.0 |
|  |  |  | 0.241 | 4.207 | 3.958 | 0.000 | -43.5 | -78.0 | 33.0 |
| 0.111 | 666 | 0.031 | 0.093 | 4.552 | 4.245 | 0.000 | 27.0 | -46.5 | -12.0 |
|  |  |  | 0.605 | 3.776 | 3.590 | 0.000 | 21.0 | -31.5 | -10.5 |
| 0.142 | 591 | 0.040 | 0.218 | 4.246 | 3.991 | 0.000 | -46.5 | -40.5 | -19.5 |
|  |  |  | 0.389 | 4.006 | 3.788 | 0.000 | -43.5 | -58.5 | -18.0 |
|  |  |  | 0.793 | 3.575 | 3.414 | 0.000 | -48.0 | -58.5 | -10.5 |
| 0.662 | 144 | 0.286 | 0.569 | 3.813 | 3.622 | 0.000 | 51.0 | -63.0 | 0.0 |
| 0.791 | 86 | 0.411 | 0.629 | 3.752 | 3.569 | 0.000 | -22.5 | -30.0 | -16.5 |
| 0.944 | 15 | 0.758 | 0.674 | 3.705 | 3.528 | 0.000 | 57.0 | -34.5 | 37.5 |
| 0.517 | 218 | 0.192 | 0.691 | 3.688 | 3.513 | 0.000 | 49.5 | -51.0 | 34.5 |
|  |  |  | 0.732 | 3.644 | 3.475 | 0.000 | 51.0 | -57.0 | 43.5 |
| 0.940 | 17 | 0.740 | 0.774 | 3.597 | 3.433 | 0.000 | 51.0 | 12.0 | 31.5 |
| 0.893 | 40 | 0.587 | 0.791 | 3.577 | 3.416 | 0.000 | -19.5 | -6.0 | 60.0 |
| 0.918 | 28 | 0.657 | 0.816 | 3.546 | 3.389 | 0.000 | 31.5 | -51.0 | 55.5 |
| 0.911 | 31 | 0.638 | 0.842 | 3.512 | 3.358 | 0.000 | 27.0 | -84.0 | 19.5 |
| 0.901 | 36 | 0.608 | 0.854 | 3.495 | 3.344 | 0.000 | -55.5 | -40.5 | -4.5 |
| 0.922 | 26 | 0.670 | 0.931 | 3.361 | 3.225 | 0.001 | 48.0 | -40.5 | -18.0 |

Table C: VBM results for regions showing a positive relationship with *L* (thresholded at *p* <.001 uncorrected, cluster size > 10 voxels).

| **Region** | **MNI coordinates (mm)** | **(BIC single) – (BIC double)** | **Posterior probability of single regressor model** | ***R* (younger)** | ***R* (older)** |
| --- | --- | --- | --- | --- | --- |
| ***ΔLL*** | | | | | |
| Left anterior prefrontal cortex | -19.5 52.5 19.5 | 2.45 | 0.92 | 0.44 | 0.42 |
| ***L*** | | | | | |
| Left posterior parietal cortex | -28.5 -79.5 39.0 | 4.27 | 0.99 | 0.27 | 0.66 |
| Left  hippocampus | -22.5 -30 -16.5 | 4.36 | 0.99 | 0.32 | 0.46 |
| Right hippocampus | 21 -30 -12 | 4.22 | 0.99 | 0.37 | 0.43 |
| Left anterior prefrontal cortex | -21 55.5 21 | 4.37 | 0.99 | 0.22 | 0.39 |

Table D: *Post hoc* analysis of VBM results for regions showing significant positive relationship with *ΔLL* or *L*. Corrected grey matter density values were extracted from the peak voxel locations indicated, and subject to Bayesian model comparison. In all regions, model comparison clearly favoured a model in which a single regressor encoded the variable of interest across age groups, suggesting a similar relationship between grey matter density and our variables of interest over the course of healthy ageing.

Supplemental Figures


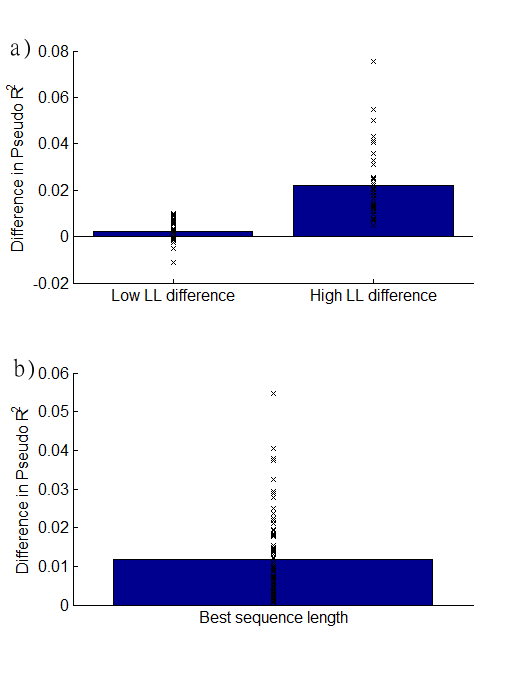


**Figure S1a: Bar plot illustrating the mean difference in pseudo *R*^2^ scores between the best sequential inference model and the filtering model. Subjects were split into two groups using a median split based on *ΔLL* (low *ΔLL* on the left, high *ΔLL* on the right).**  **As expected, the best sequential inference model resulted in a greater increase in ability to predict subjects’ behaviour in the high *ΔLL* group. This illustrates the utility of *ΔLL* as a metric of between-subject variability (bar indicates mean group values, crosses indicate individual subject values)**

**Figure S1b: Bar plot illustrating how using the best sequence length improves behavioural model fitting. This is quantified as the difference in pseudo *R*^2^ scores between using the optimal sequence length (*L*) for each subject and the average across all other sequence lengths (bar indicates mean value, crosses indicate individual subject values)**


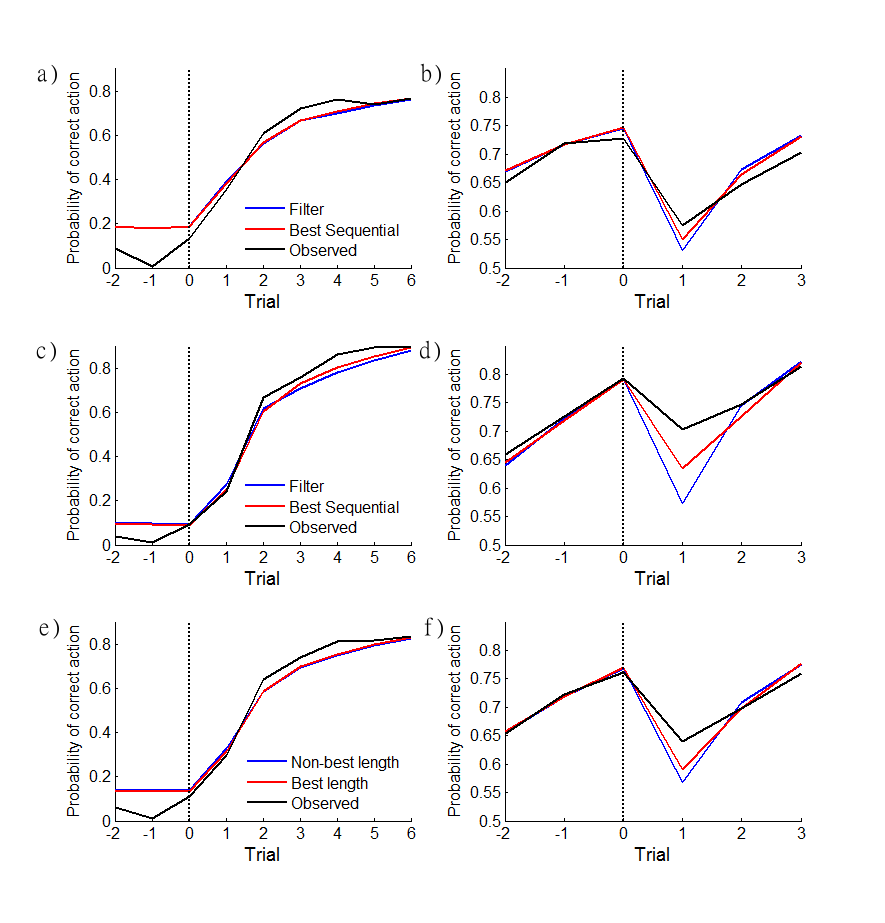


**Figure S2: Plots illustrating group level average performance of the models at predicting behavioural data averaged across reversal events (a,c,e) and misleading outcomes (b,d,f) (situations where the contingencies had not reversed, but improbable feedback was observed suggesting that they might have). The y axis indicates the probability of taking the action that is correct after the reversal in the left column of panels, and the action that is correct throughout the whole time window in the right column. The dotted line at trial 0 indicates the timing of the event itself.**

**S2a-d: Using the best sequential inference model (red) resulted in more accurate predictions of observed choice behaviour (black) than the filtering model (blue). As might be expected, these differences appear greater in the high ΔLL group (c,d) than the low ΔLL (a,b) group (subjects were split into two groups using a median split based on ΔLL).**

**S2e,f: Using the optimal sequence length for each subject (red) resulted in improved accuracy in predicting observed behaviour (black) when compared with taking the average predictions of the non-optimal models (blue).**
